# Urban foraging in Brazilian public greenspaces

**DOI:** 10.1007/s13280-023-01847-y

**Published:** 2023-03-21

**Authors:** Solène Guenat, Jonas P. Bailey-Athias, Leonie K. Fischer

**Affiliations:** 1 Institute for Landscape Planning and Ecology, University of Stuttgart, Keplerstraße 11, 70174 Stuttgart, Germany; 2 Economics and Social Sciences, Swiss Federal Institute for Forest, Snow and Landscape Research WSL, Zürcherstrasse 111, 8903 Birmensdorf, Switzerland; 3 Recife, Brazil

**Keywords:** Greenspace perceptions, Integrative landscape planning, Latin American biodiversity, Local ecological knowledge, Foraging regulations, Wild edible species

## Abstract

Urban foraging, i.e., the gathering of wild edible plants, plays a key role in nature connection within cities. Its integration in planning could contribute to the conservation of urban biodiversity. However, we have little understanding of the interactions between the motivations for and barriers to foraging, and the role of legislation, especially in biodiversity hotspots. Through an online questionnaire and policy review, we explored the practice of urban foraging in Recife, Brazil, across social, spatial and regulatory dimensions. We found that most non-foragers would forage if pollution risks were addressed and knowledge was improved. Foragers collected up to 31 species, none of which are threatened. By integrating the social, spatial and regulatory dimensions of the practice, we highlighted the importance of the local context for targeting foraging incentives. In all, regulation had little impact on where the practice is carried out, and foraging seemed to have little negative impact on biodiversity, as no threatened species were collected and foragers were conscious of their impact. This knowledge can contribute to better integrate the practice of foraging within legislation and develop forager-led greenspace planning and management. In biodiversity hotspots threatened by urban expansion, foraging can contribute to slowing down the biodiversity crisis and improve urban residents’ contact with biodiversity.

**Supplementary Information:**

The online version contains supplementary material available at 10.1007/s13280-023-01847-y.

## Introduction

Foraging is an informal, ancient practice consisting of the collection of non-timber forest products (NTFP) not explicitly planted, maintained or cultivated for consumption. It is performed worldwide in a diverse range of habitats, such as agricultural landscapes (Bharucha and Pretty 2010), forests (Pawera et al. 2020) or urban areas (Landor-Yamagata et al. 2018). Urban foraging is practiced by people from diverse socio-economic backgrounds (Schlesinger et al. 2015; Arrington et al. 2017). In the Global South, however, urban foragers are predominantly lower income residents (Mollee et al. 2017; Garekae and Shackleton 2020a; Somesh et al. 2021). Urban foraging takes place in a variety of formal and informal greenspaces, both private and public. The practice typically takes place spontaneously when a potential resource is encountered (Landor-Yamagata et al. 2018). Urban foragers tend to gather two to eighteen different plant species, including native and non-native ones (Mollee et al. 2017; Synk et al. 2017; Landor-Yamagata et al. 2018).

Urban foraging is recognised for providing many types of benefits to cities and their residents. Firstly, it helps combat urban food insecurity by providing additional cost-free food sources. African studies showed that fruits and plants foraged in urban areas can improve human diet by providing important nutrient and food diversity to local communities (Schlesinger et al. 2015; Garekae and Shackleton 2020a). In Latin America, fruit yield from street trees could fill up to 64 % of the municipal calorie deficit (Vannozzi Brito and Borelli 2020). Secondly, foraging can encourage biodiversity conservation measures within cities, especially when foragers can draw from a diversity of ecosystems with high plant species richness (Fischer et al. 2020). Plants foraged both in Germany and in the USA are often unconventional food plants (Synk et al. 2017; Landor-Yamagata et al. 2018), there being a minimal risk of overharvesting native or rare species (Fischer and Kowarik 2020). In the long run, greenspace management for foraging would thus benefit plants that are otherwise not maintained or supported as cultivated species and encourage the creation of habitat for many non-crop species. Thirdly, foraging improves the connection with nature in densely built areas where such connection is often lacking (McLain et al. 2014). Additionally, foragers often possess sophisticated local ecological knowledge (Chipeniuk 1998) and demonstrate both a stronger sense of belonging and enhanced well-being compared to non-foragers (Shackleton et al. 2017). Motivations for engaging in foraging and the derived benefits are diverse. Hereby, the provision of food is often cited, including by studies in the Global North (Poe et al. 2013; Landor-Yamagata et al. 2018). Other motivations include the enjoyment provided by the practice, the connection with nature it helps to build and the enhancement of culinary experiences (Synk et al. 2017). Foraging is also motivated by health considerations like access to, and use of, medicinal plants collected (Poe et al. 2013; Mollee et al. 2017) or by the mental and physical health benefits of the activity itself (Synk et al. 2017). In the Global South, cultural considerations and health benefits were considered even more important than contributions to livelihood, though the latter was still recognised by two thirds of foragers (Garekae and Shackleton 2020b). Common reasons cited for not engaging in foraging are the lack of time and knowledge (Synk et al. 2017; Garekae and Shackleton 2020b; Somesh et al. 2021). Improving foraging knowledge might require innovative solutions, given that such knowledge is often shared among family or close community members (Poe et al. 2013; Landor-Yamagata et al. 2018). Inadequate access to foraging sites (Somesh et al. 2021) and unfavourable regulations (Synk et al. 2017; Garekae and Shackleton 2020b) also limit the practice. Both these concerns closely relate to how foraging is encouraged with urban planning and greenspace management.

Urban foraging can also improve the planning and management of urban greenspaces, and some studies link the local practice to the legislative barriers of the investigated cities. Regulations influencing foraging revolve around nature conservation, greenspace management and park use. Despite a few exceptions in the Global North like the Forestry Management Plan for Seattle (Hurley and Emery 2018) and the “edible city” of Andernach, Germany (Kosack 2016), foraging itself is largely overlooked in urban planning. When it is recognised, it is often discouraged or considered illegal (McLain et al. 2014; Shackleton et al. 2017). Reasons for dismissal by planning authorities include risks to public health, the acceleration of biodiversity loss due to over-harvesting and damage to vegetation (Shackleton et al. 2017). Yet foraging can foster a sense of place-making, spontaneous co-management (McLain et al. 2014) and deepen sense of ownership (Hurley et al. 2015), thus promoting bottom-up collective greenspace management that provides economic gains for municipalities (Lafontaine-Messier et al. 2016). Additionally, although some found that threatened species can be foraged for non-commercial purposes (Landor-Yamagata et al. 2018), others highlighted low risks to native species as the frequency of collected NTFP reflected their abundance (Fischer and Kowarik 2020). As for concerns about risks to public health, most are related to the consumption of polluted products (Russo et al. 2017). However, studies on contamination found that plants collected along low-trafficked streets had heavy metal levels below recommended thresholds (Amato-Lourenco et al. 2020). Consequently, despite negative framings of foraging in policies, most urban land managers in South Africa can envisage the practice as a tool to improve sustainability and biodiversity conservation, as well as the safety of public greenspaces by activating their regular use (Sardeshpande and Shackleton 2020).

While we have a good understanding of the plant-related social and spatial dimensions of urban foraging, such understanding mainly emerges from studies conducted in the Global North (McLain et al. 2014; Synk et al. 2017; Landor-Yamagata et al. 2018; Fischer and Kowarik 2020) or the old world (Shackleton et al. 2017; Garekae and Shackleton 2020b; Somesh et al. 2021). We know very little about the practice in Latin America and the Caribbean, even though 81 % of this region’s population lives in urban areas (DESA 2018), with 41.9 % experiencing food insecurity (FAO et al. 2021). Additionally, there is a strong lack of understanding of how social and spatial dimensions of foraging relate to regulations in force.

With this study, we seek to explore the practice of urban foraging in a Latin American biodiversity hotspot across social, spatial and regulatory dimensions. Specifically, we investigate the motivations and concerns leading to the uptake of foraging, hypothesising that the practice is linked to gardening, growing up in a rural environment, a higher number of greenspace visits, a closer relationship with nature and a healthier diet. We also explore the places used for urban foraging, the species collected and the integration of the practice within the greenspace regulatory framework. Finally, we investigate whether this framework can impact foraging, investigating relationships between the level of area protection and the number of foragers using a specific neighbourhood for the practice. By synthesising the social, spatial and regulatory drivers leading to foraging, we aim to develop recommendations to improve the accessibility of the practice, thereby increasing the socio-environmental resilience of cities in the Global South.

## Materials and methods

### Study area

Recife is a tropical coastal city located in Northeast Brazil. It is the ninth most populous city in the country, has an estimated population of 1.45 million (Instituto Brasileiro de Geografia e Estatística 2010) and a surface area of 218.84 km^2^ (Instituto Brasileiro de Geografia e Estatística 2021). Recife is known for its extreme level of socioeconomic inequality, being the 8th most income-segregated city out of the 151 Brazilian cities (dos Santos et al. 2021).

Recife lies on an alluvial plain. A diversity of ecosystems like mangrove forests, coastal vegetation and dense ombrophilious forest are found within its borders (Sá Carneiro and de Barros Mesquita 2000; Braga et al. 2021). The dense ombrophilious forest is characteristic of the Atlantic forest biodiversity hotspot, highly threatened by urban expansion (Seto et al. 2012). As of 2014, 45.58 % of Recife’s land area is covered by greenspaces, amounting to 46m^2^ per inhabitant (de Oliveira et al. 2014). Sixty-four percent of these greenspaces consist of fragmented forests in nature reserves, with the remaining 36 % being gardens, parks and street vegetation sparsely distributed in built-up areas (de Oliveira et al. 2014). There is no data on the repartition of those greenspaces per neighbourhood, but access worldwide, including in Brazil, tends to be unequal (Rigolon et al. 2018).

### Study design

In this research, we used transdisciplinary methods to explore the social, spatial and regulatory aspects of foraging in Recife, Brazil. We developed a questionnaire-based survey to understand (1) motivations for urban foraging by exploring incentives with the potential to motivate non-foragers to take up the practice and by looking at relationships between respondents’ foraging practices and their relationship with greenspaces and gardening; and (2) how foraging is practiced, regarding places and species. For the latter, we identified food uses, plant life form and protection status through a literature review. We then conducted a policy review to investigate the extent to which the practice of foraging is acknowledged and allowed in local regulations. Finally, we used spatial analyses to link information about foraging practices ascertained from the survey with the regulations identified through the policy review (Table 1).

**Table 1:** Aims of the study and data sources

Aims	Data collection method
Investigating the motivations and concerns leading to the uptake of foraging	Questionnaire
Exploring the places used for urban foraging and the species collected	Questionnaire
Determining the species foraged	Questionnaire
Describing species uses, life form and protection status	Literature review
Presenting the impact of the greenspace regulatory framework on foraging	Policy review
Investigating the relationships between the practice of foraging and its regulatory framework	Policy review, questionnaire, spatial analysis

### Survey

The questionnaire was initially developed in English, based on Fischer and Kowarik (2020) and Mollee et al. (2017). It was then adapted to suit the local and cultural context and translated into Brazilian Portuguese. The resulting questionnaire (Table S1) consisted of 25 multiple-choice and five open-ended questions, including items exclusively for foragers (*n* = 6) and non-foragers (*n* = 2). The questionnaire included an introductory statement and six sections (Table 2), focusing on respondents’ (1) foraging behaviour; (2) gardening practices; (3) connection with greenspaces; (4) perception of their diet; (5) socio-economic background; and (6) knowledge regarding existing urban greenspace initiatives, and specific public places used for foraging. Finally, respondents could provide any additional information they thought important about foraging. The questionnaire followed the guidelines established by Brazilian federal law no. 13.709, with respect to anonymity and data privacy.

**Table 2:** Questionnaire structure

Questionnaire section	Question	
	Number	Type
1. Foraging behaviour, including whether they forage and:	1	Close-ended
1a. (For foragers) their motivations, the places the forage and the plants collected	5	Close-ended
	1	Open-ended
1b. (For non-foragers) their concerns regarding foraging	2	Close-ended
2. Practice of gardening	3	Close-ended
3. Relationship with greenspaces	3	Likert ranking
	3	Close-ended
4. Diet perception	2	Likert ranking
5. Socio-economic background: gender, age, income, education level, neighbourhood of residence, growing-up environment	6	Close-ended
	1	Open-ended
6. Existing initiatives on urban greenspaces and additional information	3	Open-ended

The questionnaire was distributed online using Google Forms between 22 April 2021 and 22 June 2021, with a mix of purposive and snowball sampling approaches. It was sent to two foragers known to the authors, with requests to forward it further. The questionnaire was also shared on Whatsapp and Facebook groups focusing on city development (69 members), architecture (59 members), urban agroecology (116 members), natural landscapes (51 members) and permaculture (554 members). Finally, it was distributed to an Urban Silviculture class (28 students) at the Federal Rural University of Pernambuco and shared on one author’s WhatsApp, Instagram, and Facebook accounts (Table S2).

### Policy review

To examine the extent to which foraging is formally acknowledged and allowed in the study area, we carried out a qualitative review of local legislation and policies that might impact foraging. The policy search was carried out between February and June 2021 and updated in April 2022. We searched Recife’s official website (Leis Municipais 2022) and websites of specific protected areas (Protection Units). Keywords were selected in Brazilian Portuguese relating to food and extraction of natural resources (for all keywords, see Table S3). To be included in the review, the documents had to be local legislation documents focusing on biodiversity conservation, environmental or land-use issues in the geographical boundaries of the city of Recife. Legislation focusing on food provisioning or food security were excluded from the review, as were any state- or national-level legislations.

### Data curation and analysis

Any respondent below 18 or residing outside of Recife’s boundary was excluded from the analysis. We used binomial generalised linear models with a probit link to analyse whether any variable had an impact on engaging in foraging. We used engagement in foraging as the response variable and the following nine explanatory variables: age, gender, education, income, childhood environment, practice of gardening, frequency of greenspace use, relationship with nature and perception of their diet (Table 3). Models were run with all possible combinations of variables and compared according to AICc, selecting the one with ΔAICc ≤ 2 (Burnham and Anderson 2002), using the R statistical package MuMIn, v.1.43.17 (Bartoń 2022). All statistical analyses were carried out with R, v.4.1.2 (R Core Team 2021).

**Table 3:** Explanatory variables included in the statistical models

Explanatory variables	Type of data	Categories
Age	Categorical	18–24; 25–34; 35–44; 45–54; 55–65; 65+
Gender	Categorical	Female, male, prefer not to say
Education	Categorical	Highest qualification achieved: no education, elementary, high school, bachelor, postgraduate
Income	Ranked	No income, less than 1 min wage, 1–2 min wage; 2–4 min wage; 4–6 min wage; 6+ min wage
Childhood environment	Categorical	Urban, rural
Practice of gardening	Categorical	Gardener, non-gardener
Frequency of greenspace use	Ranked	Several times a week, once a week, less than once a week, never, I do not know
Relationship with nature	Ranked	Score of 0 (weak connection) to 12 (strong connection)
Diet perception	Ranked	Score of 0 (unhealthy) to 8 (healthy)

Places where foraging took place and conditions necessary for foraging uptake by non-foragers were quantified by how many times each was mentioned. The number of foraging places and conditions necessary for foraging uptake cited per respondent was also quantified.

Latin names of plants were identified based on Recife’s urban forestry management plan (Secretaria de Meio Ambiente e Sustentabilidade 2013) and Lorenzi (1992). Each foraged plant was then classified according (1) its life form, namely fungus, herb or woody vegetation; (2) status as a consumable plant, either considered unconventional, i.e. plants that are edible but which are not commonly used as food; with unconventionally-used parts; or totally conventional, based on Kinupp and Lorenzi (2014), (3) origin, either native or non-native, and (4) protected status, according to the Brazilian list of protected species (Ministério de Meio Ambiente 2014) and the IUCN Red List (IUCN 2022).

Identified regulations were used to classify areas according to whether they fell under specific regulation and, if so, according to their legislation on NTFP extraction, namely (a) no mention of NTFP, (b) NTFP extraction allowed, (c) NTFP extraction allowed only for non-commercial purposes or (d) NTFP extraction not allowed. Such areas were mapped using official municipal georeferenced data together with the locations where foraging was practiced (obtained from the survey) in QGIS v.310.13 (QGIS Development Team 2021). Foraging locations and regulations frameworks were correlated by describing the number of foragers collecting plants in neighbourhoods falling under each type of regulation.

## Results

### Respondent profiles

Out of the 256 respondents, 97 (37.9 %) foraged (Table S4). More than half of the respondents were female (59 %) and the majority was under 44 years old (68.4 %). Respondents generally had a high socio-economic status. Eighty percent held a university degree and 64.1 % had a monthly income of at least four federal minimum wages (R$4.180). Most respondents were raised in urban areas (89.5 %). Greenspace use varied, with nearly half of respondents indicating that they garden (49.6 %) and visit greenspaces at least once a week (44.5 %). Most had a strong relationship with nature, with 86.3 % stating that they like spending time in nature, and that greenspaces (91.0 %) and contact with nature (81.3 %) are important to them. Similarly, respondents mostly perceived their diets as healthy (76.2 %) and ate fruits and vegetables (88.3 %).

### Drivers of foraging

**Fig. 1:**
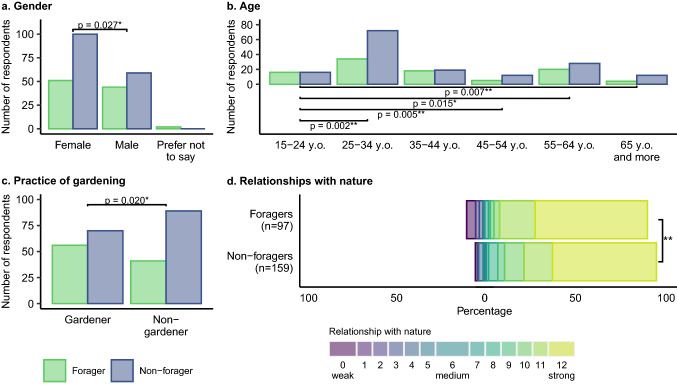
Variables significantly influencing whether a respondent forages, or not. **a** Gender, **b** age, **c** practice of gardening and **d** relationship with nature, estimated through the aggregation of the evaluation, on a 5-items Likert scale, of three different statements

Four explanatory variables were included within the single best-fit model used to identify the impact of socio-economic and nature-relation variables on engagement in foraging, all of them with a significant effect on the uptake of foraging. The model showed that foragers were more likely to be male than female (*β* = 0.401, SE = 0.181, *p* = 0.027*; Fig. 1a; Table S5), more likely to be either below 24 years old or within the 35–44 age bracket (Fig. 1b; Table S5) and more likely to be gardeners (*β* = 0.438, SE = 0.188, *p* = 0.020*) than non-foragers. Foragers were also more likely to have either a very strong (scores of 11 or 12 out of 12) or a very weak connection with nature (scores of 0 or 3 out of 12). Non-foragers, however, were more likely to have a moderately strong connection with nature (scores of 8–10 out of 12), while there were no differences between foragers and non-foragers in the likelihood of a moderately weak connection with nature (4–7 out of 12) (Fig. 1d; Table S5).

Qualitative comments from the respondents corroborated the model results. For instance, comments emphasized the relation to gardening and to the food provisioning aspect of foraging by stating how *“*[as a forager,] *I think there should be more edible plants because there are a lot of hungry people.”* Similarly, the comments substantiated foragers’ very strong connection with nature. One forager mentioned how “*green areas should be protected or given greater attention by environmental bodies.*” Specific attachment to the places was evident, with foragers describing how “*together with* [their] *neighbours*, [they] *look after a small square in front of the house.* [They] *nicknamed it “Granny Regina Square”, after* [their] *grandmother.*”

### Conditions for foraging uptake

**Fig. 2:**
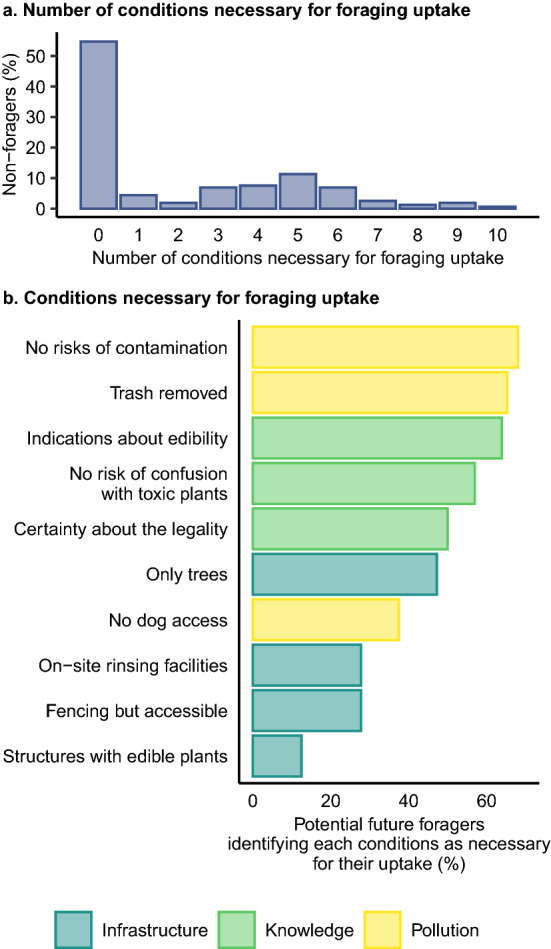
**a** Number and **b** type of conditions identified by the non-foraging respondents as necessary conditions for them to take up foraging. Values indicate the number of respondents selecting each category. Total respondent number exceed the 159 as participants were free to select several conditions

Out of the 159 non-foragers surveyed, only 23 (14.5 %) respondents mentioned that no measure could motivate them to forage, while the other 136 (85.5 %) mentioned that they might be interested in foraging under certain circumstances (Fig. 2a). Non-foraging respondents identified an average of 4.57 (± 0.24) conditions, out of a possible ten, which would need to be fulfilled for them to take up foraging, (Fig. 2a). Conditions to be fulfilled related to the natural and man-made infrastructure where foraging could potentially take place (*n* = 4), knowledge about foraging (*n* = 3) and pollution (*n* = 3). Pollution was a top concern, with the need to remove contamination risk and trash being the most frequently identified conditions necessary for the uptake of foraging (*n* = 49 and 47 respectively; Fig. 2b). Non-foragers wondered “*how* [one could] *guarantee that* [the plant] *is not poisoned*”. Regarding knowledge, non-foragers reported that learning how to identify an edible plant (*n* = 46), together with measures to reduce risk of confusion with toxic plants (*n* = 41) and improved clarity about the legality of foraging (*n* = 36), would help motivate them to start foraging.

For instance, one non-forager wrote “*Foraging? Is it allowed?*”. Some infrastructural changes like planting forageable trees rather than herbaceous vegetation (*n* = 34), building fences so that “*the collection site* [is safe]” (*n* = 20) or including rinsing facilities (*n* = 20) were also recognised, though by fewer participants (Fig. 2b).

### Places for foraging

**Fig. 3:**
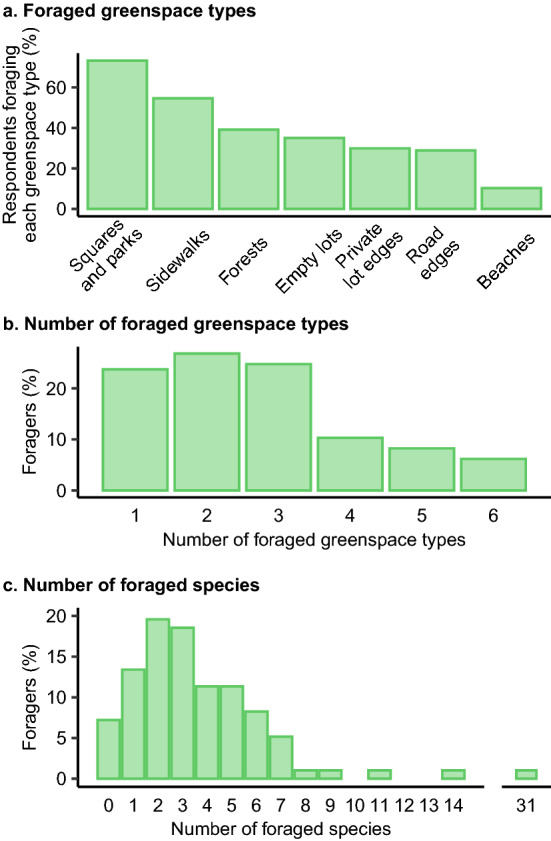
Description of the practice of foraging through the **a** type of greenspace foraged, **b** the number of greenspaces used and **c** the number of plants foraged per respondent. Values indicate the number of respondents

**Fig. 4:**
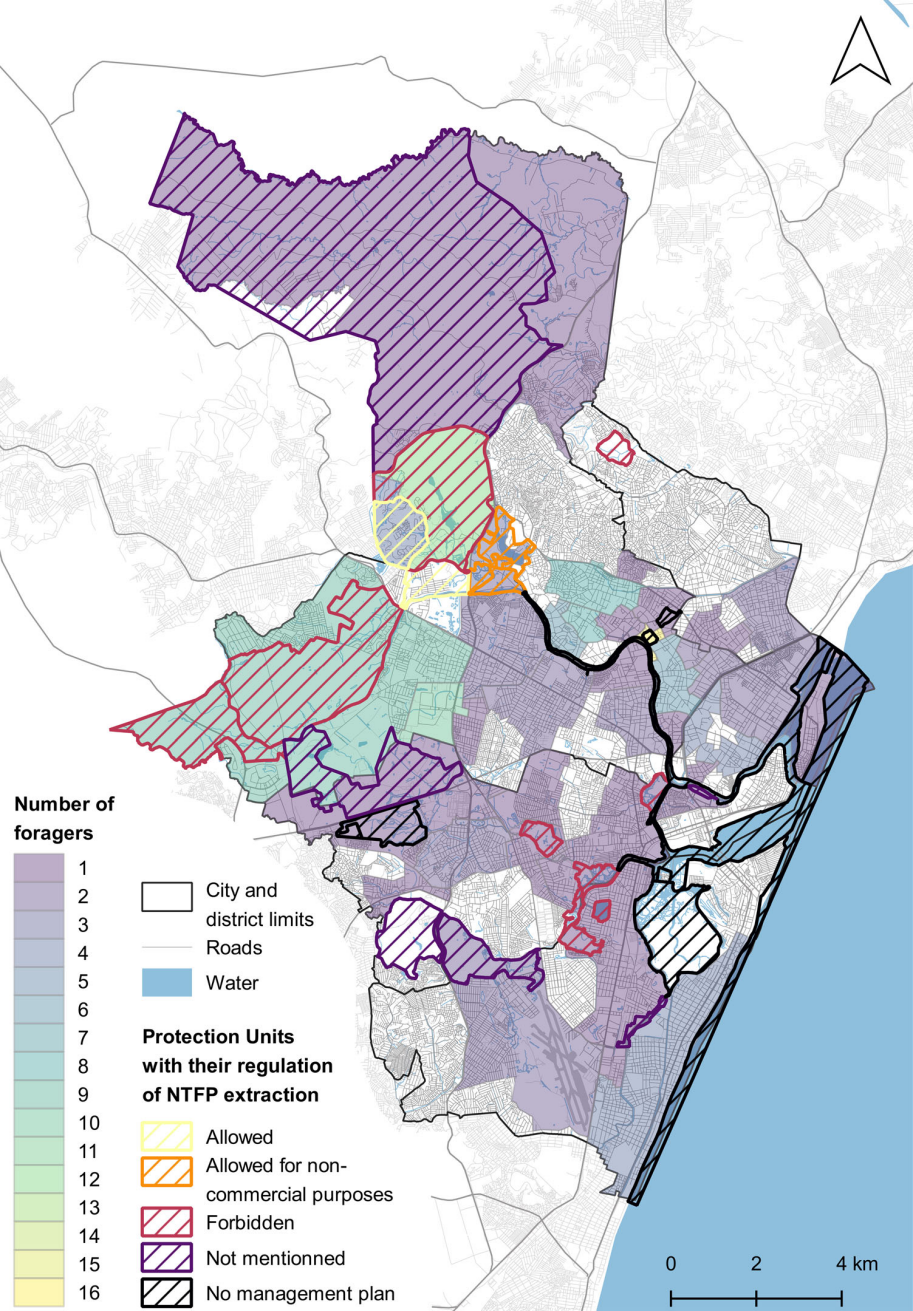
Map of Recife, including the number of respondents foraging in each neighbourhood as well as the Protection Units and their respective regulations. Areas outside of Protection Units could be foraged as part of a specific project and with approval from the municipality

Foraging respondents used an average of 2.71 (range of 0–10) different types of greenspaces for the practice (Fig. 3a). The most used greenspaces were squares and parks (*n* = 71; Fig. 3b), followed by sidewalks (*n* = 53). Other types of greenspaces foraged included forests (*n* = 38), empty lots (*n* = 34) private lot edges (*n* = 29), road edges (*n* = 28) and beaches (*n* = 10). Foraging took place in most neighbourhoods of Recife, except in the most populated neighbourhoods in the North and in the South of the city (Fig. 4). Specifically identified greenspaces included the university campus and conservation areas, such as Dois Irmãos or Várzea in the Northwest (Fig. 4). Riverside neighbourhoods were also mentioned as foraging sites, though to a lesser extent (Fig. 4).

Concerns were raised from both foragers and non-foragers about the environmental impact of the practice on greenspaces. For instance, a non-forager stated how they “*would like people to be satisfied with taking advantage of the many social and environmental services of green areas without resorting to foraging that may compromise their integrity*.” Some foragers also experienced changes about the state of urban greenspaces across time, mentioning how “*the foraging I am talking about took place in public spaces when I was a child. Today there are no more fruit trees in good shape*.”

### Species of interest

**Fig. 5:**
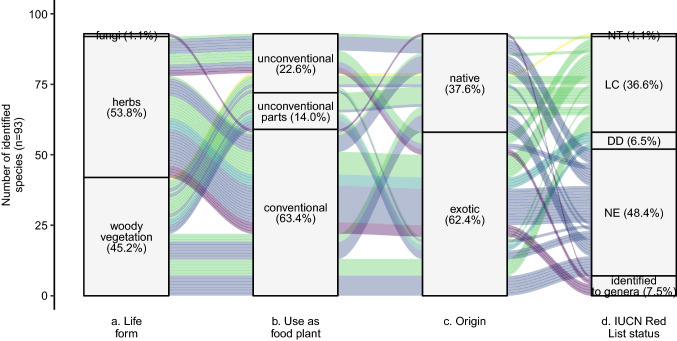
Number and proportion of the identified foraged species according to **a** their life form, **b** whether they are conventionally considered as conventional food or not, **c** their origin and **d** their status on the IUCN Red List. Colours follow the Red-List status. *NT* near threatened in yellow, *LC* least concern in green, *DD* data deficient in turquoise, *NE* not evaluated in blue; and plants identified to genera (no IUCN red list available) in violet

A total of 93 species from 49 different families were foraged (Table S6). Foragers mentioned collecting an average of 3.7 species (range 0–31; Fig. 3c). All species collected except one were plants, the exception being a basidiomycete fungi. Plants were divided nearly equally between herbs and woody vegetation (53.8 % and 45.2 % respectively; Fig. 5a). Most foraged plants were fruit-bearing (61.6 %), such as the rose-apple (*Syzygium malaccense* L.). However, 22.6 % of the collected species are considered unconventional food plants. This includes the Surinam cherry (*Eugenia uniflora* L.), which was the third most commonly foraged species. Partly unconventional food plants made up 14.0 % of all foraged species and included the most collected (the mango, *Mangifera indica* L.), whose leaves were foraged by one respondent (Fig. 5b). About a third of the collected plant species are native to Brazil (Fig. 5c). While more than two thirds of the conventional food plants are non-native species (71.2 %), there was a more equal distribution of non-native and native species within the unconventional food plants (Fig. 5c). Of the foraged plants, none are on the list of Brazilian’s protected species and only one (the pink trumpet tree, *Handroanthus impetiginosus* (Mart. ex DC.) Mattos) is considered near threated on the IUCN Red List. This is a native woody species, not conventionally used as a food plant. However, evaluation of the threatened status is limited, as many species are categorised by the Red List as "data deficient” (6.5 %) or “not evaluated” (48.8 %), and a few could not be identified to the species level (7.5 %; Fig. 5d).

### Regulation of foraging

**Fig. 6:**
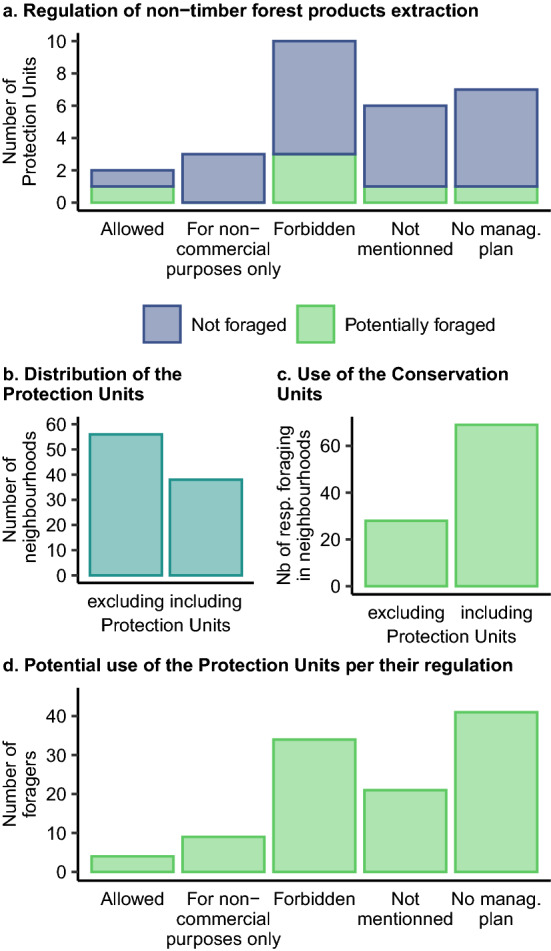
Regulation, distribution and use of the Protection Units. **a** Number of Protection Units depending on their regulation of NTFP extraction and whether they were foraged; **b** spatial distribution of the Protection Units in Recife’s neighbourhoods, **c** potential use of Protection Units for foraging and **d** potential use of the Protection Units for foraging units according to their regulation. Total number of respondents exceed the number of foragers as respondents, in addition to having to select one neighbourhood, had the possibility to mention additional foraging spaces

We identified a total of 31 municipal laws and decrees, including eleven laws and one decree with jurisdiction covering the entire city of Recife (Table S7) and 19 decrees governing protected areas only (Fig. 4; Table S8). The twelve laws and decrees regulating greenspaces within Recife focused on themes such as land-use and zoning, standards for sidewalks, protection against climate change, creation of a sustainability strategy and urban afforestation. Particularly relevant was Municipal Law No. 17.367 which mandates that at least 40 % of all planted trees within the city should be fruit trees, thus indirectly promoting foraging practices. This law, however, restricts the planting of edible species to certain greenspaces, limiting their use as street trees due to the potential harm of falling fruits like mangoes to people, private and public properties and infrastructure. The same law regulates the use of fruit tree species with edible value, mandating a permit for plant collection. Jurisdiction for handing out permits falls within the municipality and permits will only be granted if foraging is part of a specific project. No city-wide regulation was found regarding the use of edible herbs or mushrooms, however Municipal Law No. 18.014 establishes a municipal system of protected units of greenspaces. This system of protected units aims to strategically manage and restore all urban blue-green networks within the city limits, focusing on improving human health and well-being, promoting biodiversity, and addressing climate change. This law creates the basis for Conservation Units, which are directed by specific decrees and should provide a management plan. Such Protection Units cover 38 % of the area of Recife and are included in 37 of Recife’s 94 neighbourhoods (Fig. 6b). Conservations Units are divided in four categories, namely botanical gardens, nature conservation units, landscape conservation units and environmental balance units.

We identified management plans for 21 of the 28 Protection Units (Fig. 4). These 21 Protection Units with management plans were either classified as Nature Conservation Units (*n* = 18) or Landscape Conservation Units (*n* = 3). According to Municipal Law No. 18.014, use of the land, e.g. for building or agriculture, is allowed within these Protections Units. The main differences between Nature and Landscape Conservation Units are their size and the inclusion of “specimens of the local or regional biota, whose natural attributes justify their protection and conservation, in view of their ecological relevance” within Landscape Conservation Units (Municipal Law 18.014, art. 18.II). The degree of restrictions based on this categorisation would allow foraging of NTFPs for both commercial and non-commercial purposes. However, tougher regulations can be established within management plans. Within the 21 management plans considered, most (*n* = 10) entirely forbid the extraction of NTFP productions, three allow it only for non-commercial purposes and two allow it without restrictions (Fig. 6a). Six management plans did not mention NTFP extraction (Fig. 6a).

### Relationships between the regulation and practice of foraging

About a third of foragers (34.6 %) foraged in neighbourhoods without any Protection Units (Fig. 6b). These are areas where fruits trees can legally be foraged only as part of a municipality-approved project. Out of the 65.4 % respondents who foraged in neighbourhoods with Protection Units, most foraged in neighbourhoods including Protection Units without management plans (*n* = 41) or in areas where NTFP extraction was explicitly forbidden (*n* = 34) (Fig. 6d). However, neighbourhoods under both these jurisdictions are also the most common (Fig. 6a). There was no foraging reported in neighbourhoods with Protection Units allowing NTFP extraction only for non-commercial purposes (Fig. 6a).

## Discussion

By exploring the rarely investigated practice of urban foraging within a biodiversity hotspot of Latin America, we gained insights that can help support the practice in its legal framing, and by extension improve city dwellers’ connection with nature and biodiversity conservation within cities. Our interdisciplinary study highlights new opportunities for encouraging foraging in Latin America. Nearly all non-foragers declared being ready to take up the practice given specific conditions, such as reduced pollution, improved foraging knowledge and clearer legislation. The practice was carried out in a diversity of greenspaces, where usually two to three species were collected by foragers. From the legal perspective, foraging was discouraged in most of the city even though the planting of edible fruit trees was mandated. Protection Units, covering 38 % of the city area, included specific regulations either allowing or forbidding foraging. Our novel approach, jointly analysing the social, spatial and regulatory dimensions of foraging, highlighted the low impact of legislation, as areas in which foraging was allowed were not more foraged than other greenspaces.

About a third of respondents identified as foragers, a proportion consistent with that found in Berlin (Fischer and Kowarik 2020). This number might be an underestimation, given that the survey took place when access to public greenspace was restricted due to the COVID-19 pandemic. This foraging rate, however, shows that Recife has not yet reached urban densities high enough to significantly hinder interactions with nature, as is the case in India where only 16 % of the population forage (Somesh et al. 2021). Conversely, foraging rates were not high enough to suggest that the practice is widely necessary for sustenance, as in African countries where foraging rates are above 50 % (Kaoma and Shackleton 2015; Schlesinger et al. 2015; Mollee et al. 2017; Garekae and Shackleton 2020b). In our study, foragers were most likely to be young males who garden and express either a very weak or a very strong connection with nature. They did not significantly differ from non-foragers according to their childhood environment, current income, education level, how often they visited greenspaces or how they perceived their diets. Consequently, their socio-economic characteristics did not match those of European foragers, as neither age, gender nor gardening were significant in studies conducted in Berlin and Vienna (Fischer and Kowarik 2020; Schunko and Brandner 2022). Urban foragers in Brazil also differed from African, Indian and North American patterns where generally more women foraged (Arrington et al. 2017; Garekae and Shackleton 2020b; Somesh et al. 2021) and from the USA where older people tended to forage more (Arrington et al. 2017). As such, we highlight that there is no universal profile of foragers. Even visits to greenspaces, which could have been understood as a precondition or consequence of the practice and is highly significant in Europe (Fischer and Kowarik 2020), was not related to foraging engagement in this study. This lack of a universal forager-profile highlights the importance of understanding the diversity of local contexts for urban foraging.

It is often stated that foraging increases environmental awareness and connection with nature (McLain et al. 2014; Synk et al. 2017; Schunko and Brandner 2022). However, we observed that non-foragers sometimes criticised the practice for its impact on the integrity of greenspaces. Additionally, our results do not fully corroborate this biospheric perspective, as some foragers exemplified a very strong connection with nature, but others a very weak connection. Yet connection with nature can take many forms, including utilitarian views, which are relatively prevalent among foragers (Schunko and Brandner 2022). These views may have been shared by the foragers that are identified in our study as having a weak connection to nature. The potential relationship between weak connection to nature in our study and utilitarian views of nature is reinforced by foragers’ engagement with gardening, where plants are specifically cultivated to be used. Similarly to gardening, foraging relies on knowledge of urban nature, highlighting a strong awareness component of the connection, not necessarily captured by our survey. This knowledge base was critical, as foragers identified a total of 93 species, including as many as 31 per forager. Local foraging knowledge is often acquired through family or community members (Landor-Yamagata et al. 2018) and by participating in the practice since childhood (Chipeniuk 1998). Foragers also demonstrated knowledge of the change of the landscape and of plant abundances. We therefore emphasise the need for inclusion of local ecological knowledge in urban foraging incentives.

Governance of greenspace and foraging was identified as mainly a top-down process. Greenspace planting and extraction was regulated through a diversity of municipal laws mandating city officials for their implementation. Planting of edible trees was encouraged, as is often the case in Brazil (Vannozzi Brito and Borelli 2020). However, the practice of foraging itself was discouraged by the requirement of permits. Such patterns are common across the globe, with foraging being discouraged, forbidden (McLain et al. 2014; Shackleton et al. 2017) or not recognised in most plans (Clark and Nicholas 2013). However, environmental managers in some regions value the practice for its impact on crime reduction (e.g., by attracting people to otherwise empty greenspaces), promotion of biodiversity conservation measures (Sardeshpande and Shackleton 2020) and the removal of fallen fruits (McLain et al. 2014).

One reason often cited by legislators for not allowing foraging is its impact on the environment (Shackleton et al. 2017). Cities contain higher proportions of threatened species than their surrounding landscapes (Ives et al. 2016) and foraging can put protected species at risk under certain circumstances (Molnár et al. 2017; Petersen et al. 2012). Despite uncertainties regarding the threatened status of many collected species, only one out of 93 plants foraged was on the IUCN Red List. This plant, *H. impetiginosus*, is near threatened due to logging in its natural environment (Hills 2021), but often planted for ornamental purposes in Brazilian urban areas. Such a low number of threatened species collected is consistent with other studies showing little impact of foraging on threatened taxa (Fischer and Kowarik 2020; Landor-Yamagata et al. 2018). Usually, diverse landscapes are preferred by foragers, as they enable easy collection and limit the risk of over-exploitation (Brandner and Schunko 2022). Preferences for diverse landscapes could explain the dominance of parks as foraging grounds. Protecting large and diverse greenspaces to incentivise foraging would equally benefit other plant species, as between 17 and 68 % of all plant species found in cities are non-edible (Hurley and Emery 2018; Nero et al. 2018), and their associated fauna.

Prohibition of foraging within most protection units did not hinder the practice. Foraging was carried out at similar rates throughout the city, irrespectively of the legislation. Foraging without legal protection is relatively common (Lee and Garikipati 2011). Consequently, encouraging forager-led cooperative resource management is more likely to ensure the sustainability of the practice (Lee and Garikipati 2011) and improve cities’ economic benefits (Lafontaine-Messier et al. 2016) as opposed to banning it altogether. Our study respondents freely reported instances where they self-organised greenspace implementation and management. Consequently, there is potential in Recife for supporting foraging-led community involvement in urban greenspace planning and management. This could, in the long term, improve the city’s sustainability and biodiversity conservation through a bottom-up approach. The legal aspects would, however, need to be adapted and clarified, particularly within the many protection units without management plans or regulations regarding foraging.

Clarification and communication of the legislation may encourage foraging. Most non-foraging respondents showed an interest in taking up the practice, similar to results from other contexts (Fischer and Kowarik 2020; Nero et al. 2018). Non-foragers identified specific conditions that need to be fulfilled before they would consider foraging, including increased knowledge about its legality. Main concerns, however, related to the toxicity and pollution of foraged products, an issue commonly highlighted as a barrier by non-foragers (Brandner and Schunko 2022; Fischer and Kowarik 2020; Landor-Yamagata et al. 2018) and as a public health risk by legislators (Russo et al. 2017). Foraging was found to mostly take place in squares and parks, as is the case in North America (Poe et al. 2013) and Europe (Fischer and Kowarik 2020; Landor-Yamagata et al. 2018). Yet studies on plant contamination within urban areas show that proximity to highly-trafficked roads is the key factor in increasing plant contamination, with pollution levels decreasing rapidly the further their distance from such roads (Amato-Lourenco et al. 2020; Antisari et al. 2015). Foragers tend to be aware of this fact and select traffic-free foraging sites (Brandner and Schunko 2022). Yet in our study, sidewalks were the second-most foraged greenspace type, though we did not consider traffic density. Observed reductions in pollution levels with increasing distance from highly-trafficked roads highlight the potential for foraging safely within the city limits. Communication measures could encourage existing foragers to differentiate between sites and inform the wider population of the potentially lower risk of pollution away from the road.

## Conclusion

Through this transdisciplinary study of urban foraging in a biodiversity hotspot of the Global South, we highlight that, given the diversity of foragers across countries, no specific group can be targeted to promote foraging worldwide. Incentives to motivate the high proportion of urban residents with interest in the practice need to focus on universal themes, such as addressing urban pollution and improving knowledge transmission about plants, their use, their associated risks and foraging-relevant legislation. We show high engagement levels coupled with important local ecological knowledge, which needs to be integrated in assessments of the dynamics of urban biodiversity. In our case study, foraging did not threaten local biodiversity despite common perceptions. Conversely, it has the potential to increase urban biodiversity protection by adjusting greenspace management measures. These could incentivise foraging and be developed as participatory management practices to include foragers’ strong attachment to and community involvement towards greenspaces. However, the current greenspace governance structure does not encourage foraging despite recognising benefits in the study area. Integration of forager-led approaches to greenspace planning and management within the regulatory framework would support urban sustainability and cities contributions towards the fight against the biodiversity crisis.

## Supplementary Information

Below is the link to the electronic supplementary material.

Supplementary file1 (PDF 5072 KB)
