# Supplementary material for: Urban foraging in Brazilian public greenspaces
Source: Ambio. 2023 Mar 21;52(7):1248–61. doi: 10.1007/s13280-023-01847-y (PMC10029783; doi:10.1007/s13280-023-01847-y)
Supplement: Supplementary file 1 — Supplementary file1 (PDF 5072 KB) [file 13280_2023_1847_MOESM1_ESM.pdf]

Ambio

Supplementary Information
This supplementary information has not been peer reviewed.

Title: **Urban foraging in Brazilian public greenspaces**

Authors: S. Guenat[1,2]*, J.P Bailey-Athias[1,3] and L.K. Fischer[1]

[1]Institute for Landscape Planning and Ecology, University of Stuttgart, Keplerstraße 11, 70174, Stuttgart, Germany
[2]Economics and Social Sciences, Swiss Federal Institute for Forest, Snow and Landscape Research WSL, Zürcherstrasse 111, 8903 Birmensdorf, Switzerland
[3]Recife, Brazil
*corresponding author: solene.guenat@wsl.ch

Table S1. English version of the questionnaire distributed

Section	Text	Possible answers
0. Introduction	Your participation is voluntary and confidential. This survey is in accordance with Brazil's federal law No. 13.709, the General Law of Data Protection, which establishes definitions regarding personal data, sensitive data, control, processing, consent, anonymization, etc.	I declare that I have read and agree to participate. I accept that you treat my data only for research purposes
1. Foraging behaviour	1.1 Have you ever collected edible plants outside private plots in the city?	a. Yes, but only a few times<br>a. Yes, occasionally<br>a. Yes, quite often<br>b. No, never
	1.2.a Which edible or medicinal plants have you already harvested in public areas?	[open question]
	1.3.a How do you use edible plants that you (occasionally) harvest off private plots?	Fruits and nuts<br>Herbs and spices<br>Raw or fresh<br>Cooked or dehydrated<br>Tea<br>Jam<br>Medicine<br>Pet food<br>Religious rituals<br>Others, specify:
	1.4.a In what kind of space do you collect edible plants?	Sidewalks<br>Squares and parks<br>Empty lots<br>Private lot edges<br>Road edges<br>Forests<br>Beaches<br>Community gardens<br>Other, specify
	1.5.a In which neighbourhood do you gather or have you gathered the most?	[list of Recife's 94 neighbourhoods]
	1.6.a Did you engage in urban foraging in public spaces during the COVID-19 pandemic?	Yes, about the same as usual<br>Yes, less than usual<br>Yes, more than usual<br>No
	1.7.a Have you or would you market what you collect?	Yes<br>No

	1.2.b Would you collect edible plants from public green spaces?	Yes, without constraints (redirected to Q2.1)<br>Yes, with constraints<br>No (redirected to Q2.1)
	1.3.b Would you have any restrictions regarding the collecting of edible plants in public greenspaces	Areas without dog access<br>Site is fenced but accessible<br>If there is signs indicating edible plants<br>If the trash is removed regularly<br>Structures like flower beds are found<br>If the gathered plants can be rinsed on site<br>If the products can be harvested from trees<br>There are no concerns about potential contaminants<br>Only if I am aware of the legality<br>If the plants cannot be mistaken with toxic on[illegible]<br>Other, specify:
2. Gardening practice	2.1 Do you garden	a. Yes<br>b. No
	2.2.a Where do you mostly practice gardening	At work<br>Balcony<br>In my garden/backyard<br>In a community garden<br>Other, specify:
	2.3.a For what reason do you not practice gardening?	No interest<br>I would like to, but I have no space<br>I would like, but I have no financial conditions<br>I would like to, but I do not have time<br>I would like to, but I do not know where<br>I would like to, but I do not know how<br>Other, specifically:
3. Relationship with greenspaces	3.1 To what extend do the following statements apply to you?<br>I like to spend my free time in a natural environment.<br>Green spaces near me are important to me.<br>Contact with nature is always important to me.	(for each statement)<br>Disagree<br>Partially disagree<br>Indifferent<br>Partially agree<br>Agree
	3.2 How often do you usually visit a public greenspace in the city?	Several times a week<br>Once a week<br>Less than once a week<br>Never<br>I do not know

	3.3 Are you in favour of planting more edible plants in public green areas in the city?	a. Yes<br>b. No
	3.4.b What is the main reason for you to be against, the increased planting of edible species in public areas?	Risk of damage to people and property, due to falling fruit<br>Possible pest control problem, because fruits attract rodents and insects<br>Possible harvest disputes"<br>Possible more maintenance<br>Other, specify:
4. Relationship with food	4.1 To what extend do the following statements apply to you?<br>I like to eat fresh fruit and vegetables.<br>In general, I think I have a healthy diet	(for each statement)<br>Disagree<br>Partially disagree<br>Indifferent<br>Partially agree<br>Agree
5. Socioeconomic background	5.1 Gender	Female<br>Male<br>Prefer not to say
	5.2 What is your age group?	up to 18 years<br>19 to 24 years<br>25 to 34 years<br>35 to 44 years<br>45 to 54 years<br>55 to 64 years<br>65 or more
	5.3 What is your occupation/type of work	[open entry]
	5.4 What is your monthly income	I have no monthly income<br>Less than 1 minimum wage (R$ 1.100,00)<br>Between 1 minimum wage to 2 minimum wages<br>Between 2 to 4 minimum wages<br>Between 4 to 6 minimum wages<br>More than 6 minimum wages
	5.5 What is your level of education?	No Education<br>Complete elementary education<br>Incomplete elementary education<br>Complete high school<br>Incomplete high school<br>Completed bachelor's degree<br>Incomplete bachelor<br>Postgraduate studies

	5.6 In which neighbourhood do you live?	[list of Recife's 94 neighbourhoods and "I do not live in Recife, but in the metropolitan area"]
	5.7 In which environment did you mainly grow up?	Urban<br>Rural
6. Additional information (optional)	6.1 Do you know of any organisation or initiative in the city that promotes urban agriculture? If yes, which?	[open entry]
	6.2 Is there a specific public place or locations that you have already collected that you would like to share (example: name of a square, street)?	[open entry]
	6.3 Is there anything else important that we have not discussed about collection practices or anything else you would like to add?	[open entry]

Table S2. Social media groups targeted for the distribution of the online survey, with their reach.

Platform	Group	Reach
Whatsapp	INCITI – Pesquisa e Inovação para as Cidades	69
	Universidade Federal de Pernambuco Arquitetura	59
	RMR Urban Agroforestry	116
	Associação Águas do Nordeste (NGO)	51
Facebook	Universidade Federal de Pernambuco	63 000
	Permacultura Pernambuco	554

Table S3. Keywords used for the policy review search, in their original (Brazilian Portuguese) and translated version

Keywords used, in Brazilian Portuguese	English translation
Alimento	Aliment
Alimentação	Alimentation
Comida	Food
Comestível	Edible
Fruta/o	Fruit
Frutífera/Fruteira	Fruit tree
Extrativismo	Extractivism
Extração	Extraction
Não-Madeireiros	Non-timber forest products

Table S4. Distribution of the respondents' answers in regards to their relationship with nature and diet, in relation with their practice of foraging.

Survey questions	Variables	Foragers (n=97)	37.89%	Non-foragers (n=159)	62.11%
Gender	Female	51	52.58%	100	62.89%
	Male	44	45.36%	59	37.11%
	Prefer not to say	2	2.06%	0	0.00%
Age	15-24 years old	16	16.49%	16	10.06%
	25-34 years old	34	35.05%	72	45.28%
	35-44 years old	18	18.56%	19	11.95%
	45-54 years old	5	5.15%	12	7.55%
	55-64 years old	20	20.62%	28	17.61%
	More than 64 y.o.	4	4.12%	12	7.55%
Income	No monthly income	4	4.12%	7	4.40%
	Less than 1 min. wage (R$1100)	4	4.12%	2	1.26%
	1-2 min. wage	14	14.43%	15	9.43%
	2-4 min. wage	20	20.62%	26	16.35%
	4-6 min. wage	19	19.59%	40	25.16%
	More than 6 min. wage	36	37.11%	69	43.40%
Education	No education	2	2.06%	0	0.00%
	Elementary school	1	1.03%	1	0.63%
	High school	22	22.68%	24	15.09%
	Bachelor	29	29.90%	54	33.96%
	Postgraduate	43	44.33%	80	50.31%
Childhood environment	Rural	10	10.31%	17	10.69%
	Urban	87	89.69%	142	89.31
Gardening	Yes	57	57.73%	70	44.03%
	No	42	42.27%	89	55.97%
Frequency of greenspace visits	Several times a week	25	25.77%	20	12.66%
	Once a week	28	28.87%	41	25.95%
	Less than once a week	38	39.18%	74	46.84%
	Never	1	1.03%	10	6.33%
	I do not know	5	5.15%	14	8.86%
Would consider foraging	Without constraints	NA	NA	64	40.25%
	With constraints	NA	NA	72	45.28%
	Never	NA	NA	23	14.47%
Orientation towards nature					
I like to spend my free time in a natural environment	Agree (4)	67	69.07%	102	64.15%
	Partially agree (3)	18	18.56%	34	21.38%
	Indifferent (2)	1	1.03%	12	7.55%
	Partially disagree (1)	3	3.09%	8	5.03%
	Disagree (0)	8	8.25%	3	1.89%
Greenspaces near me are important to me	Agree (4)	82	84.54%	131	82.39%
	Partially agree (2)	4	4.12%	16	10.06%
	Indifferent (2)	2	2.06%	5	3.14%
	Partially disagree (1)	4	4.12%	4	2.52%

	Disagree (0)	5	5.15%	3	1.89%
Contact with nature is always important to me	Agree (4)	74	76.29%	123	77.36%
	Partially agree (3)	13	13.40%	21	13.21%
	Indifferent (2)	0	0.00%	10	6.29%
	Partially disagree (1)	4	4.12%	3	1.89%
	Disagree (0)	6	6.19%	2	1.26%
Nature orientation score	0 (weak connection)	5	5.15%	2	1.26%
	1	0	0.00%	0	0.00%
	2	2	2.06%	0	0.00%
	3	2	2.06%	1	0.63%
	4	0	0.00%	4	2.52%
	5	1	1.03%	8	5.03%
	6 (medium connection)	0	0.00%	3	1.89%
	7	2	2.06%	2	1.26%
	8	1	1.03%	8	5.03%
	9	2	2.06%	6	3.77%
	10	3	3.09%	17	10.69%
	11	19	19.59%	25	15.72%
	12 (strong connection)	60	61.86%	91	57.23%
Diet					
I like to eat fresh fruits and vegetables	Agree (4)	71	73.20%	123	77.36%
	Partially agree (3)	13	13.40%	19	11.95%
	Indifferent (2)	3	3.09%	6	3.77%
	Partially disagree (1)	4	4.12%	6	3.77%
	Disagree (0)	6	6.19%	5	3.14%
In general, I think I have a healthy diet	Agree (4)	38	39.18%	58	36.48%
	Partially agree (3)	34	35.05%	65	40.88%
	Indifferent (2)	10	10.31%	10	6.29%
	Partially disagree (1)	10	10.31%	16	10.06%
	Disagree (0)	5	5.15%	10	6.29%
Diet score	0 (unhealthy)	5	5.15%	3	1.89%
	1	0	0.00%	2	1.26%
	2	3	3.09%	4	2.52%
	3	4	4.12%	7	4.40%
	4 (medium)	3	3.09%	8	5.03%
	5	6	6.19%	10	6.29%
	6	11	11.34%	13	8.18%
	7	29	29.90%	57	35.85%
	8 (healthy)	36	37.11%	55	34.59%

Table S5. Parameter estimates, standard error, z-values and p-values for the single best-fitted model (DeltaAICc≤2) for engaging in foraging.

	Estimate	Std. Error	z value	Pr(r(>|z|)
(Intercept)	1.131	0.607	1.863	0.062
Age				
25-34 years old	-0.967	0.306	-3.156	0.002**
35-44 years old	-0.498	0.354	-1.404	0.160
45-54 years old	-1.264	0.453	-2.789	0.005**
55-64 years old	-0.826	0.340	-2.430	0.015*
More than 64 y.o.	-1.263	0.467	-2.707	0.007**
Gender				
Prefer not to say	6.110	412.788	0.015	0.988
Male	0.401	0.181	2.215	0.027*
Gardening	0.439	0.188	2.332	0.020*
Nature connection score				
2	4.908	378.821	0.013	0.990
3	0.358	0.933	0.384	0.701
4	-6.373	283.882	-0.022	0.982
5	4.831	605.102	0.008	0.994
6	-6.653	299.827	-0.022	0.982
7	-0.303	0.834	-0.363	0.717
8	-2.201	0.794	-2.771	0.006**
9	-1.483	0.749	-1.981	0.048*
10	-2.108	0.667	-3.159	0.002**
11	-0.904	0.565	-1.601	0.109
12	-1.017	0.534	-1.905	0.057

N=284. *P<0.05; **P<0.01; ***P<0.001.

Table S6. Species collected and their attributes

Family	Species	Common name (English)	Common name (Portuguese)	IUCN Red List status	Life form	Origin	Use as food plant	Nb. of foragers
Acanthaceae	*Justicia gendarussa* Brum.f.	Willow-leaved justicia	Abre-Caminho	Not evaluated	Herb	Non-native	Conventional	1
Amaranthaceae	*Amaranthus* spp.	Slender Amaranth	Caruru/Bredo-Do-Mato/Amaranto	Identification not precise enough	Herb	Non-native	Unconventional	2
	Dysphania ambrosioides (L.) Mosyakin & Clemants	Jesuit's Tea/Mexican-Tea	Mastruz	Not evaluated	Herb	Native	Conventional	7
Anacardiaceae	*Anacardium occidentale* L.	Cashew	Caju	Least concern	Woody	Native	Conventional	6
	Mangifera indica L.	Mango, Mango leaves	Manga/ Manga Espada	Data deficient	Woody	Non-native	Unconventional parts	55
	Schinus terebinthifolius G. Raddi	Brazilian Peppertree	Aroeira/Aroeira-Da-Praia	Not evaluated	Woody	Native	Conventional	11
	Spondias mombin L.	Yellow Mombin	Cajá	Least concern	Woody	Native	Unconv. parts	12
	Spondias purpurea L.	Red Mombin	Ciriguela	Least concern	Woody	Native	Unconv. parts	1
	Spondias tuberosa Arruda	Brazil Plum	Umbu	Least concern	Woody	Native	Unconv. parts	1
Annonaceae	*Annona squamosa* L.	Sugar Apple, Sugar Apple leaf	Pinha, Folha de Pinha	Least concern	Woody	Native	Unconventional parts	2
Apiaceae	*Pimpinella anisum* L.	Anise	Erva Doce	Not evaluated	Herb	Non-native	conventional	3
Apocynaceae	*Plumeria rubra* L.	Frangipani	Jasmin	Least concern	Herb	Non-native	Unconventional	1
Araceae	*Colocasia esculenta (L.) Schott*	Taro	Inhame Roxo	Least concern	Herb	Non-native	Conventional	1

	Xanthosoma sagittifolium (L.) Schott	Arrowleaf Elephant Ear	Taioba	Not evaluated	Herb	Native	Unconventional	2
Arecaceae	*Elaeis guineensis* Jacq.	African oil palm	Dendê	Least concern	Woody	Non-native	Conventional	2
	Euterpe oleracea Mart.	Açaí palm	Açaí	Not evaluated	Woody	Native	Unconventional parts	1
	Mauritia flexuosa L.f.	Moriche Palm	Buriti	Not evaluated	Woody	Native	Conventional	1
Asphodelaceae	*Aloe vera* (L.) Brum.f.	Aloe Vera	Babosa	Not evaluated	Herb	Non-native	Conventional	6
Asteraceae	*Acmella oleracea* (L.) R.K.Jansen	Toothache plant	Flor de Jambu	Not evaluated	Woody	Native	Unconventional	1
	Artemisia vulgaris L.	Mugwort	Artemísia	Least concern	Herb	Non-native	Conventional	1
	Baccharis trimera (Less.) DC.	Carqueja	Carqueja	Not evaluated	Herb	Native	Conventional	1
	Cynara scolymus L.	Artichoke	Alcachofra	Not evaluated	Herb	Non-native	Conventional	1
	Galinsoga parviflora Cav.	Gallant Soldier	Picão Branco (Guasca)	Not evaluated	Herb	Non-native	Unconventional	1
Basidiomycota			Basidiomycete mushroom	Identification not precise enough	Fungi	Native	Conventional	1
Bigoniaceae	*Handroanthus impetiginosus* (Mart. ex DC.) Mattos	Trunk Of Pink Trumpet Tree	Casca de Ipê Roxo	Near threatened	Woody	Native	Unconventional	1
Bixaceae	*Bixa orellana* L.	Achiote	Urucum	Least concern	Woody	Native	Unconventional	1
Brassicaceae	*Brassica oleracea* L.	Wild cabbage	Couve	Data deficient	Herb	Non-native	Conventional	1
Cactaceae	*Pereskia aculeata* Mill.	Blade-Apple Cactus	Ora-Pro-Nobis	Least concern	Herb	Native	Unconventional	2

Caricaceae	*Carica papaya* L.	Papaya and leaves	Mamão/ Folhas de Mamão	Data deficient	Woody	Non-native	Unconventional parts	2
Caryophyllaceae	*Dianthus caryophyllus* L.	Carnation	Cravos	Not evaluated	Herb	Non-native	Conventional	1
Chrysobalanaceae	*Licania tomentosa* (Benth.) Fritsch.	Not Found	Oiti	Not evaluated	Woody	Native	Conventional	2
Cocosaceae	*Cocos nucifera* L.	Coconut Tree	Coco	Not evaluated	Woody	Non-native	Conventional	1
Cucurbitaceae	*Cucurbita* spp.	Pumpkin, leaves Of Pumpkin	Jerimum/ Folhas De Jerimum	Identification not precise enough	Herb	Non-native	Conventional	1
	Momordica charantia L.	Carilla gourd	Melão de São Caetano	Not evaluated	Herb	Non-native	Conventional	1
Euphorbiaceae	*Cnidoscolus aconitifolius* (Mill.) I.M.Johnst.	Chaya/Spinach Tree	Chaya, Shaya	Least concern	Woody	Non-native	Unconventional	1
	Jatropha gossypiifolia L.	bellyache bush	Pinhão Roxo	Not evaluated	Herb	Native	Conventional	2
	Ricinus communis L.	Castor Bean	Mamona/ Carrapateira	Least concern	Herb	Non-native	Unconventional	2
Fabaceae	*Bauhinia forficata* Link	cow's-foot	Pata de Vaca	Least concern	Woody	Native	Unconventional	1
	Caesalpinia pyramidalis Tul.		Catingueira	Least concern	Woody	Native	Conventional	1
	Prosopis juliflora (Sw.) DC.	Mesquite	Algaroba	Not evaluated	Woody	Non-native	Conventional	1
	Senna occidentalis L.	Stinking weed	Fedegoso/Mangerioba	Least concern	Herb	Native	Conventional	1
	Stryphnodendron barbadetimam (Vell.) Mart.	Stryphnodendron	Barbatimão	Not evaluated	Woody	Native	Conventional	1
	Tamarindus indica L.	Tamarind	Tamarindo	Least concern	Woody	Non-native	Conventional	6

	Trifolium spp.	Clover	Trevos	Identification not precise enough	Herb	Non-native	Unconventional	1
Hibisceae	*Hibiscus rosa-sinensis* L.	Hibiscus	Hibiscos	Not evaluated	Herb	Non-native	Conventional	2
Lamiaceae	*Melissa officinalis* L.	Lemon Balm	Erva Cidreira	Least concern	Herb	Non-native	Conventional	9
	Mentha spicata L.	Mint	Hortelã	Least concern	Herb	Non-native	Conventional	12
	Ocimum basilicum L.	Basil	Manjericão	Not evaluated	Herb	Non-native	Conventional	10
	Ocimum gratissimum L.	Clove basil	Alfavaca de Caboclo	Not evaluated	Herb	Non-native	Conventional	2
	Plectranthus barbatus Andrews	Forskohlii	Tapete de Oxalá	Not evaluated	Herb	Non-native	Conventional	2
	Salvia rosmarinus Spenn.	Rosemary	Alecrim	Least concern	Herb	Non-native	Conventional	1
	Stachys byzantina K.Koch	Lamb's-Ear	Peixinho	Not evaluated	Herb	Non-native	Unconventional	1
Lauraceae	*Cinnamomum verum* J.Presl	cinnamon	Canela	Not evaluated	Woody	Non-native	Conventional	1
	Laurus nobilis L.	Bay laurel	Folha de Louro	Least concern	Herb	Non-native	Conventional	1
	Persea americana Mill.	Avocado	Abacate	Least concern	Woody	Non-native	Unconventional parts	2
Lythraceae	*Cuphea carthagenensis* (Jacq.) J.F.MacBr.	Colombian waxweed	Pé-de-pinto	Not evaluated	Herb	Native	Unconventional	1
	Punica granatum L.	Pomegranate	Romã	Least concern	Woody	Non-native	Conventional	1

Malpighiaceae	*Malpighia emarginata* DC.	Barbados Cherry	Acerola	Not evaluated	Woody	Non-native	Unconventional parts	18
Malvaceae	*Pachira aquatica* Aubl.	Malabar chestnut	Monguba	Not evaluated	Woody	Native	Conventional	1
Melastomaceae	*Miconia crenata* (Vahl) Michelang.	Soapbush	Caiuia	Not evaluated	Herb	Native	Conventional	1
Meliaceae	*Azadirachta indica* A.Juss	Neem	Nim	Least concern	Woody	Non-native	Conventional	1
Monimiaceae	*Peumus boldus* Molina	Boldo	Boldo	Least concern	Herb	Non-native	Conventional	5
Moraceae	*Artocarpus heterophyllus* Lam.	Jackfruit	Jaca	Not evaluated	Woody	Non-native	Conventional	3
	Morus nigra L.	Black Mulberry and leaves	Algaroba	Not evaluated	Woody	Non-native	Unconventional parts	4
Musaceae	*Musa* spp.	Banana	Banana	Identification not precise enough	Herb	Non-native	Conventional	2
Myrtaceae	*Eugenia pyriformis* Cambess	Uvaia	Ubaia/Uvaia	Least concern	Woody	Native	Conventional	1
	Eugenia uniflora L.	Surinam Cherry, Surinam Cherry leaves	Pitanga, Folhas de Pitanga	Least concern	Woody	Native	Unconventional	22
	Psidium cattleianum Sabine	Strawberry Guava	Araça	Not evaluated	Woody	Native	Conventional	1
	Psidium guajava L.	Guava	Goiaba	Least concern	Woody	Native	Unconventional parts	10
	Syzygium jambolanum (L.) Skeels.	Java Plum	Azeitona/Azeitona-Roxa/ Jamelão	Not evaluated	Woody	Non-native	Conventional	13
	Syzygium malaccense L.	Rose-Apple	Jambo	Least concern	Woody	Non-native	Conventional	31

Oxalidaceae	*Averrhoa carambola* L.	Star Fruit, Star Fruit leaves	Carambola/Folha de Carambola	Not evaluated	Woody	Non-native	Unconventional parts	5
Passifloraceae	*Turnera subulata* Sm.	White Buttercup	Chanana	Not evaluated	Herb	Native	Unconventional	1
Phyllanthaceae	*Phyllanthus niruri* L.	Stonebreaker	Quebra Pedra	Not evaluated	Herb	Native	Conventional	1
Piperaceae	*Peperomia pellucida* Kunth	Pepper Elder	Lingua de Sapo	Not evaluated	Herb	Native	Unconventional	1
	Piper umbellatum L.	Not Found	Capeba	Not evaluated	Herb	Native	Unconventional	1
Plantaginaceae	*Plantago major* L.	Broadleaf Plantain	Tanchagem	Least concern	Herb	Non-native	Unconventional	1
Poaceae	*Cymbopogon citratus* (DC.) Stapf.	Lemon Grass	Capim Santo	Not evaluated	Herb	Non-native	Conventional	15
Portulaceae	*Portulaca oleracea* L.	Common Purslane	Beldroega	Least concern	Herb	Non-native	Unconventional	3
Rosaceae	*Rosa* spp.	Rose	Rosas	Identification not precise enough	Herb	Non-native	Conventional	1
Rubiaceae	*Morinda citrifolia* L.	Great Morinda	Noni	Not evaluated	Woody	Non-native	Unconventional	1
Rutaceae	*Citrus × latifolia* (Yu.Tanaka) Tanaka	Persian Lime	Limao	Not evaluated	Woody	Non-native	Conventional	2
	Citrus × sinensis (L.) Osbeck	Orange	Laranja	Not evaluated	Woody	Non-native	Conventional	1
	Ruta graveolens L.	Common Rue	Arruda	Least concern	Herb	Non-native	Conventional	2
Sapindaceae	*Talisia esculenta* Radik.	Pitomba	Pitomba	Least concern	Woody	Native	Unconv. Parts	1
Sapotaceae	*Manilkara zapota* (L.) P.Royen	Sapodilla	Sapoti	Least concern	Woody	Non-native	Conventional	2
Solanaceae	*Capsicum* spp.	Pepper	Pimenta	Identification not precise enough	Herb	Non-native	Conventional	1

	Solanum lycopersicum L.	Tomato	Tomate	Not evaluated	Herb	Non-native	Conventional	2
	Solanum lycopersicum var. cerasiforme	Cherry Tomatoes	Tomate Cereja	Not evaluated	Herb	Non-native	Conventional	2
Urticaceae	*Pilea microphylla* (L.) Liebm.	Rockweed	Brilhantina	Not evaluated	Herb	Native	Conventional	1
Zingiberaceae	*Alpinia zerumbet* (Pers.) B.L.Burtt and R.M.Sm.	Shell Ginger	Colônia	Data deficient	Herb	Non-native	Conventional	5
	Curcuma longa L.	Turmeric	Açafrão	Data deficient	Herb	Non-native	Conventional	1
	Zingiber officinale Roscoe	Ginger	Gengibre	Data deficient	Herb	Non-native	Conventional	1

Table S7. Regulations governing greenspaces, their planning and their use in the city of Recife

Regulation type	Decree number	Date	Name in Portuguese	Scope
Municipal law	15.072	1988		Authorizes the executive power to declare areas as being of municipal heritage, prohibiting any tree cutting, as necessary for their preservation by their location, rarity, beauty or seed holder condition
Municipal law		1990	Lei Orgânica do Município do Recife	Defines the jurisdiction of the Municipality of Recife
Municipal law	16.243	1996	Código do Meio Ambiente e do Equilíbrio Ecológico da Cidade do Recife	Establishes the environmental policy of the City of Recife and consolidates its environmental legislation
Municipal law	16.680	2001		Provides the municipal forestation plan
Municipal law	16.890	2004		Establishes the standards for the construction, installation and maintenance of public footpaths
Municipal decree	20.604	2004		Consolidates the rules on construction, maintenance and recovery of public sidewalks or sideways.
Municipal law	17.367	2007		Mandates that, during tree planting, at least 40% are fruit trees
Municipal law	17.511	2008	Revisão do Plano Diretor do Município do Recife. Altera a Lei 16.176/1996 -Lei de Uso e Ocupação do Solo	Revision of the masterplan for Recife
Municipal law	17.666	2010		Regulate urban afforestation
Municipal law	18.011	2014		Provides a policy for sustainability and for combating climate change
Municipal law	18.014	2014		Sets up the Municipal System of Protected Units, which now considers the arborisation of the road-sides as an environmental balance unit integrated in the protected units of Recife
Municipal law	18.111	2015		Defines limits and compensation mechanisms around city squares and parks

Table S8. Regulation of non-timber forest products (NTFP) extraction within Recife's *Protection Units*

Conservation Unit name	Neighbourhood(s)	Ecosystem	Protection Units category	Decree number	Date	Management plan	Regulation of NTFP extraction
Açude De Apipucos	Apipucos	Forest	Nature Conservation Unit - Area of Relevant Ecological Interest	33.729	10/06/2020	Available	Allowed for non-commercial purposes only
Beberibe	Guabiraba, Pau Ferro and Dois Irmãos	Forest	Nature Conservation Unit - Area of Relevant Ecological Interest	35.051	05/11/2021	Available	Not mentioned
Campo Do Jiquiá	Jiquiá	Transition	Nature Conservation Unit - Area of Relevant Ecological Interest	33.733	12/06/2020	Available	Not allowed
Caxangá	Caxangá	Transition	Nature Conservation Unit - Environmental Protection Area	33.675	20/05/2020	Available	Allowed
Curado	Curado	Managed	Nature Conservation Unit - Area of Relevant Ecological Interest	33.805	17/07/2020	Non-existent	NA
Das Capivaras	Apipucos	Transition	Nature Conservation Unit - Area of Relevant Ecological Interest	33.677	20/05/2020	Available	Allowed for non-commercial purposes only
Dois Irmãos	Dois Irmãos	Forest	Nature Conservation Unit - Area of Relevant Ecological Interest	35.05	05/11/2021	Available	Not allowed
Dois Unidos	Dois Unidos	Forest	Nature Conservation Unit - Area of Relevant Ecological Interest	33.812	20/07/2020	Available	Not allowed
Engenho Uchôa	Ibura	Forest	Nature Conservation Unit - Environmental Protection Area	17.548	20/12/1996	Available	Not mentioned
Estuário do Rio Capibaribe	Afogados, Boa Vista, Brasília	Mangrove	Nature Conservation Unit	23.809	23/07/2008	Non-existent	NA

	Teimosa, Cabanga, Coelhos, Cordeiro, Derby, Graças, Ilha do Leite, Ilha do Retiro, Ilha Joana Bezerra, Imbiribeira, Iputinga, Jaqueira, Madalena, Monteiro, Paissandu, Poço da Panela, Pina, Santana, Santo Amaro Santo Antonio, Recife, São José and Torre						
Ilha Do Zeca	Joana Bezerra	Restinga	Nature Conservation Unit - Area of Relevant Ecological Interest	33.821	22/07/2020	Available	Not allowed
Iputinga	Apipucos	Transition	Nature Conservation Unit - Area of Relevant Ecological Interest	34.005	25/09/2020	Available	Allowed for non-commercial purposes only
Jardim Botânico do Recife	Curado	Managed	Botanical garden			Non-existent	
Joana Bezerra	Joana Bezerra	Mangrove	Nature Conservation Unit - Area of Relevant Ecological Interest	34.033	07/10/2020	Available	Not mentioned
Lagoa Do Araçá	Imbiribeira	Mangrove	Nature Conservation Unit - Area of Relevant Ecological Interest	34.015	30/09/2020	Available	Not allowed
Mata Da Váreza	Várzea	Forest	Nature Conservation Unit - Area of Relevant Ecological Interest	33.723	08/06/2020	Available	Not allowed

Mata Das Nascentes	Várzea	Forest	Nature Conservation Unit - Area of Relevant Ecological Interest	33.824	24/07/2020	Available	Not allowed
Mata Do Barro	Barro	Forest	Nature Conservation Unit - Area of Relevant Ecological Interest	33.861	05/08/2020	Available	Not mentioned
Matas Do Curado	Curado	Forest	Nature Conservation Unit - Area of Relevant Ecological Interest	33.827	27/07/2020	Available	Not mentioned
Orla Marítima	Boa Viagem, Brasilia Teimosa, Pina, Recife	Mangrove	Nature Conservation Unit - Area of Relevant Ecological Interest	35.512	01/04/2022	Non-existent	NA
Parque Da Tamarineira	Tamarineira	Forest	Landscape Conservation Units	34.113	06/11/2020	Non-existent	NA
Parque da Jaqueira	Jaqueira		Nature Conservation Unit	17.610	2010	Non-existent	NA
Parque dos Manguezais	Pina	Mangrove	Nature Conservation Unit	25.565	01/12/2010	Non-existent	NA
Rio Jordão	Imbiribeira and Boa Viagem	Mangrove	Nature Conservation Unit - Area of Relevant Ecological Interest	33.801	15/07/2020	Available	Not mentioned
São Miguel	Afogados	Mangrove	Nature Conservation Unit - Area of Relevant Ecological Interest	33.832	29/07/2020	Available	Not allowed
Sítio dos Pintos	Sítio dos Pintos	Forest	Nature Conservation Unit - Environmental Protection Area	33.676	20/05/2020	Available	Allowed
Sítio Grande	Imbiribeira	Mangrove	Nature Conservation Unit - Area of Relevant Ecological Interest	33.839	31/07/2020	Available	Not allowed
Tamandaré	Areias	Mangrove	Nature Conservation Unit - Area of Relevant Ecological Interest	33.848	03/08/2020	Available	Not allowed
